# Contemporary surgical management of synchronous colorectal liver metastases

**DOI:** 10.12688/f1000research.10324.1

**Published:** 2017-04-28

**Authors:** Danielle Collins, Heidi Chua

**Affiliations:** 1 Department of Colon and Rectal surgery, Mayo Clinic, Rochester, MN, USA

**Keywords:** colorectal cancer, colorectal liver metastases, synchronous, liver first, reverse strategy

## Abstract

Historically, the 5-year survival rates for patients with stage 4 (metastatic) colorectal cancer were extremely poor (5%); however, with advances in systemic chemotherapy combined with an ability to push the boundaries of surgical resection, survival rates in the range of 25–40% can be achieved. This multimodal approach of combining neo-adjuvant strategies with surgical resection has raised a number of questions regarding the optimal management and timing of surgery. For the purpose of this review, we will focus on the treatment of stage 4 colorectal cancer with synchronous liver metastases.

## Introduction

### History and epidemiology

Approximately 15–20% of patients with colorectal cancer will present with metastatic liver disease ^1, 2^, with a median survival of 8–12 months in untreated patients ^3^. On average, 20% of patients with colorectal liver metastases (CLM) will be suitable candidates for surgical resection ^4, 5^. Surgical excision of CLM has been shown to improve survival ^6– 10^. Indeed, a median survival of 3.6 years and a disease-free survival of 15.9 months are achievable ^11^. Presently, there are no strict criteria defining the resectability of liver metastases. The fundamental principle of liver metastasectomy is an ability to obtain an R0 resection while leaving sufficient remnant liver ^12^. The old teaching of 1 cm margin, fewer than four unilobar metastases, and absence of extra-hepatic disease is now outdated ^13, 14^. Current indications are removal of all deposits with an adequate clear margin and preservation of a functioning remnant of at least 30% of the total volume of the liver ^15, 16^. Limitations in resectability can be circumvented by performing multiple resections in addition to hybrid procedures combining surgery with interventional radiological techniques such as transarterial chemoembolization (TACE), portal vein embolization, or radiofrequency ablation ^17^.

The 1990s heralded a more aggressive surgical approach in the management of stage 4 colorectal cancer. Initially, CLM resection was performed only following resection of the primary tumor. Patients would first undergo colonic or rectal surgery followed by adjuvant chemotherapy ^10, 18^. If their disease did not progress on chemotherapy then they would be considered suitable candidates for liver resection. Early results demonstrated improved survival for patients who made it to liver resection; however, it was noted that a substantial proportion of patients (up to 50% in one study) did not complete this treatment course because of either delays in starting chemotherapy or interim progression to unresectable liver disease.

In realizing the pitfalls associated with this pathway and with the understanding that it is the liver disease that may be ultimately fatal (in the absence of a symptomatic primary), other strategies to deal with CLM were devised. Combined liver and colorectal resections (so-called synchronous resection) proved to be a feasible option in selected patients ^19^, with similar outcomes to the classical approach.

In 2006, a so-called “reversed” strategy of liver-targeted systemic chemotherapy followed by CLM resection and subsequent resection of the primary tumor was introduced ^20^.

This article aims to review the current literature and help clarify the complex decision-making process in the management of stage 4 colorectal cancer.

## Classical approach: treat primary first

The classical approach to patients presenting with CLM involves resection of the primary tumor followed by chemotherapy and subsequent liver resection approximately 3–6 months later. A concern with this approach is progression of liver disease in the early postoperative period or a delay in treatment owing to postoperative complications from the initial colorectal surgery. Reported rates for interval disease progression vary. In a study of 21 patients who had delayed hepatectomy, 43% (nine patients) had interval progression of their liver disease ^21^. In the multi-institutional LiverMetSurvey study, only 30% of patients managed to complete the pathway and undergo both colorectal and liver resections ^22^. However, advocates of this approach would suggest that interval progression of liver metastases is characteristic of aggressive disease biology and therefore allowing a period of time to see if there is progression avoids performing extensive hepatic resections in those who will not benefit. Indeed, the data demonstrate poorer survival in patients with early recurrence (less than 6 months following liver resection) ^23^.

## Synchronous resection

The advantage of a synchronous resection is a one-stage procedure for the patient. This has benefits in terms of planning adjuvant chemotherapy as well as cost effectiveness and shorter overall hospital stay ^24^. Obviously not all patients are suitable for a combined approach. A multidisciplinary international consensus published last year concluded that a one-stage or simultaneous resection can be carried out when the hepatectomy is not a major hepatectomy (resection of three or more liver segments) ^25^. Interestingly, this group felt that synchronous resection was more risky than separate resections. It has been hypothesized that prolonged hepatic pedicle clamping (Pringle maneuver) promotes transient ischemia, intestinal venous congestion, and bacterial translocation, which may affect the integrity of anastomoses as well as predispose to postoperative sepsis ^26^.

A meta-analysis of 22 studies with a total of 4,494 patients over the last 13 years found no significant difference in mortality or morbidity in patients undergoing synchronous resections. However, the data from these studies represent retrospective pooled results from multiple single-institution studies (with likely highly selected patients). What is coming to light is that the type of colorectal operation is important ^27, 28^. A recent NSQIP analysis categorized low-risk colorectal resections (right hemicolectomy, left colectomy without diversion, and low anterior resection) and high-risk resections (left colectomy with diversion, total abdominal colectomy, total abdominal proctocolectomy, and abdominoperineal resection [APR]), demonstrating higher morbidity and mortality in the high-risk group ^29^. In this study, the authors subdivided synchronous resections into four categories based on high-risk or low-risk liver and colorectal resections, respectively. Of 922 undergoing synchronous resection, the overall major morbidity rate was 29%. There was a trend toward higher morbidity (55% for high-risk colorectal and major hepatectomy versus 25% for low-risk colorectal and minor hepatectomy). Overall, this study demonstrates that synchronous resection is safe in patients undergoing any type of colorectal combined with a minor liver resection (RR 0.98 for morbidity and 0.28 for mortality). No definitive conclusion could be drawn regarding a synchronous high-risk colorectal resection combined with major liver resection because of the low number of patients in this cohort. A comparative study from Korea reported on 55 patients who underwent simultaneous major liver resection for CLM ^30^. They concluded that combined major liver and colorectal resection was feasible but only in highly selected patients. The reported overall morbidity was much higher in the group who had combined rectal and major liver resection, although the rate of major morbidity was similar when compared to the group who underwent staged resections. In a survey of European colorectal and liver surgeons, some had concerns regarding the postoperative morbidity from combined major liver and complex colonic procedures ^31^. Consensus from an international expert forum cautions against combined major liver and colorectal resection ^26^.

There have been several systematic reviews and meta-analyses in relation to synchronous versus staged resections. Short-term outcomes (morbidity and mortality) are similar between the two techniques ^32, 33^. There is a trend towards reduced length of stay in the synchronous group. Long-term outcomes in terms of overall survival and disease-free survival are in the region of 44% for 5-year survival with disease recurrence rates of 54–74% ^34^.

## Reverse strategy: liver first

The liver-first approach comprises three to six cycles of pre-operative liver-directed chemotherapy followed by liver resection and finally resection of the primary tumor as a staged procedure. It was first reported in 2006 ^35^. Overall, 5-year survival is in the range of 40–50% with disease-free survival of 68% at 1 year and 30% at 3 years ^23, 36^. Following the introduction of effective chemotherapeutic regimes in metastatic colorectal cancer (FOLOX, FOLFIRI), survival can be greatly improved with response rates of 40–50% following chemotherapy ^37^. Indeed, these regimens can control systemic disease, eliminate micrometastatic disease, downsize liver and primary disease, and even render the unresectable resectable. However, controversy exists as to whether pre-operative chemotherapy is required at all (in cases where liver disease is resectable). The EORTC trial, published in the Lancet in 2008 ^38^, compared neo-adjuvant FOLFOX versus up-front liver resection with no adjuvant chemotherapy in patients with resectable liver metastases. They found that patients had a longer progression-free survival in the chemotherapy group but there was no difference in overall survival. One of the flaws of this study was that patients in the “up-front” group did not get postoperative chemotherapy. The real question is whether patients should have up-front liver resection followed by adjuvant chemotherapy. An attempt to answer this question from a systematic review of recent trials did not come to any conclusion, rather advising that current studies are too heterogeneous and underpowered ^39^. However, both the NICE guidelines (UK) and the NCCN (US) recommend 6 months of perioperative systemic chemotherapy. The Charisma trial is currently recruiting in an attempt to answer this question ^40^.

The liver-first approach may prove especially useful in metastatic rectal cancer, as interval progression of liver disease can occur while patients are undergoing pre-operative radiotherapy. Furthermore, primary chemotherapy may also be of benefit in reducing the symptoms of primary disease.

## Predictors of poor response/early recurrence

Approximately 60% of patients will develop recurrence within the first 2 years following CLM resection ^7^. Several scoring systems have been devised to predict patients with poor outcomes. The Fong score ^41^ is commonly used and comprises five prognostic variables: a node-positive primary, <12 months’ disease-free interval from primary to metastases, >1 liver lesion, the largest liver lesion >5 cm, and a CEA of >200 ng/m. A score of 2 or less predicts favorable outcomes, whereas a score of 3–5 is predictive of early failure or recurrence. Early recurrence was noted in 10% of almost 6,000 resections in the LiverMetSurvey study ^42^ and conferred a much poorer overall 5-year survival of 26%. This group identified several risk factors for early recurrence, including T3–4 primary, synchronous CRLM, >3 CRLM, 0 mm margin, and intraoperative radiofrequency ablation. Early recurrence risk was reduced by adjuvant chemotherapy, a response to pre-operative chemotherapy and the use of intra-operative ultrasonography. Several factors therefore likely to contribute to early recurrence include poor pre-operative disease control and inadequate CRLM resection. Patients who do recur early may be candidates for re-resection, which does confer a survival benefit.

## Advice for management

The most important decision-making factor in the management of this complex process is to identify patients who would be suitable candidates for CLM resection. As the criteria for resectability are not fixed and are tailored to individual patients, multidisciplinary discussion is extremely important to ensure that patients are not denied a chance for curative surgery ^43^. There is evidence to suggest that multidisciplinary team discussion improves outcomes ^44^. In addition, surgical quality is important. Trained colorectal surgeons and trained hepatobiliary surgeons should be performing their respective parts of the operations in order to obtain the best outcomes. One of the difficulties in analyzing the current literature is that many studies are not based on an intention-to-treat process. Although the outcomes are similar in patients who make it to resection, there is little information on those who don’t complete the entire treatment pathway. Two recent studies suggest that 15–30% of patients may fail to complete the intended treatment ^45, 46^.

## Conclusion

There is no doubt that surgery offers the best long-term prognosis in patients with metastatic colorectal cancer. The approach to these patients has rapidly evolved over the last 30 years in combination with advances in chemotherapy, targeted molecular therapy, and superior surgical technique. The difficulty in decision making relates to the fact that the majority of the current data is retrospective in nature, with very few studies performed on an intention-to-treat basis. There are no randomized studies comparing surgery with chemotherapy or comparing different surgical techniques. Given the vast amount of literature we now have on this subject, it may be unethical to randomize outcomes at this stage.

The management of stage 4 colorectal cancer is a great example of how we can challenge and push the boundaries of medical cure in a multi-disciplinary setting.
